# Myricitrin Ameliorates Hyperglycemia, Glucose Intolerance, Hepatic Steatosis, and Inflammation in High-Fat Diet/Streptozotocin-Induced Diabetic Mice

**DOI:** 10.3390/ijms21051870

**Published:** 2020-03-09

**Authors:** Do Yeon Kim, Sang Ryong Kim, Un Ju Jung

**Affiliations:** 1 Department of Food Science and Nutrition, Pukyong National University, 45 Yongso-ro, Nam-gu, Busan 48513, Korea; dy.kim@kist.re.kr; 2 School of Life Sciences, BK21 plus KNU Creative BioResearch Group, Kyungpook National University, 1370 San-Kyuk Dong, Puk-Ku, Daegu 41566, Korea; srk75@knu.ac.kr

**Keywords:** myricitrin, diabetes, hyperglycemia, glucose intolerance, hepatic steatosis, inflammation

## Abstract

To test the hypothesis that myricitrin (MYR) improves type 2 diabetes, we examined the effect of MYR on hyperglycemia, glucose intolerance, hepatic steatosis, and inflammation in high-fat diet (HFD) and streptozotocin (STZ)-induced type 2 diabetic mice. Male C57BL/6J mice were randomly divided into three groups: non-diabetic, diabetic control, and MYR (0.005%, *w*/*w*)-supplemented diabetic groups. Diabetes was induced by HFD and STZ, and MYR was administered orally for 5 weeks. Myricitrin exerted no significant effects on food intake, body weight, fat weight, or plasma lipids levels. However, MYR significantly decreased fasting blood glucose levels, improved glucose intolerance, and increased pancreatic β-cell mass compared to the diabetic control group. Myricitrin administration also markedly increased glucokinase mRNA expression and activity as well as lowered glucose-6-phosphatase and phosphoenolpyruvate carboxykinase mRNA expression and activity in the liver. In addition, liver weight, hepatic triglyceride content, and lipid droplet accumulation were markedly decreased following MYR administration. These changes were seemingly attributable to the suppression of the hepatic lipogenic enzymes—fatty acid synthase and phosphatidate phosphohydrolase. Myricitrin also significantly lowered plasma MCP-1 and TNF-α levels and the mRNA expression of hepatic pro-inflammatory genes. These results suggest that MYR has anti-diabetic potential.

## 1. Introduction

Type 2 diabetes is characterized by impaired insulin secretion and activity [1], leading to multiple metabolic abnormalities including hyperglycemia in fasted and postprandial states. The liver is an insulin-sensitive tissue that plays a central role in the maintenance of glucose homeostasis by regulating the interaction between glucose utilization and gluconeogenesis [2]. Dysregulation of hepatic glucose metabolism plays a crucial role in the pathogenesis and complications of diabetes [1]. Insulin resistance also affects hepatic lipid metabolism through several mechanisms, including the promotion of *de novo* lipogenesis in the liver, and insulin resistance is strongly associated with the development of non-alcoholic fatty liver disease (NAFLD) [3]. Type 2 diabetes and NAFLD commonly co-exist and can synergistically cause deleterious changes [4]. Therefore, abnormal hepatic glucose and lipid metabolism are attractive therapeutic targets for type 2 diabetes.

Myricitrin (MYR) is a flavonol glycoside found in the bark of *Myrica esculenta* Buch.-Ham. ex D.Don (Myricaceae) and the leaves of *Myrica gale* L. (Myricaceae) and other plants that have been shown to possess anti-inflammatory and antioxidant activities [5]. MYR ameliorates hyperglycemia-induced oxidative stress and increases glucose uptake, glycogen content, and glucose transporter 4 gene expression in C2C12 mouse skeletal muscle cells [6]. MYR also improves diabetic cardiomyopathy in streptozotocin (STZ)-induced type 1 diabetic mice by inhibiting inflammation, oxidative stress, and apoptosis [7]. MYR also protects carbon tetrachloride-intoxicated mice against liver damage through several mechanisms that involve its antioxidant, anti-inflammatory, and anti-fibrotic activities [8]. However, the effects of MYR on hepatic glucose and lipid regulation in animal models of type 2 diabetes are not fully understood to date. Although its aglycone myricetin has been suggested to have potential for the treatment of type 2 diabetes [9,10,11], direct absorption of the glycosylated form and a higher gastrointestinal stability of MYR than myricetin were reported [12,13,14]. Therefore, it is required to evaluate the effects of MYR on type 2 diabetes.

Several non-genetic mouse models have been used to gain insight into the mechanisms of type 2 diabetes and investigate the efficacy of therapeutic candidates. Mice fed a high-fat diet (HFD) and administered STZ—a pancreatic β-cell-specific cytotoxin—are among the most widely used models of type 2 diabetes, because they closely mirror the metabolic characteristics observed in patients with type 2 diabetes including insulin resistance and reduced β-cell mass [15]. The effects of various glucose-lowering drugs, such as metformin and thiazolidinedione, in this HFD-fed STZ-induced type 2 diabetes mouse model have been confirmed [16,17]. We hypothesized that MYR would protect against type 2 diabetes in mice. To test the hypothesis, we evaluated whether MYR supplementation could improve hyperglycemia, dyslipidemia, and NAFLD in HFD/STZ-induced type 2 diabetic mice. We also investigated the effects of MYR on the changes in hepatic glucose and lipid metabolism, pancreatic β-cell mass, and inflammation to elucidate potential mechanisms of action.

## 2. Results

### 2.1. Effects of MYR on Body Weight, Fat Weight, Food Intake, and Plasma Lipid Levels

**Figure 1:**
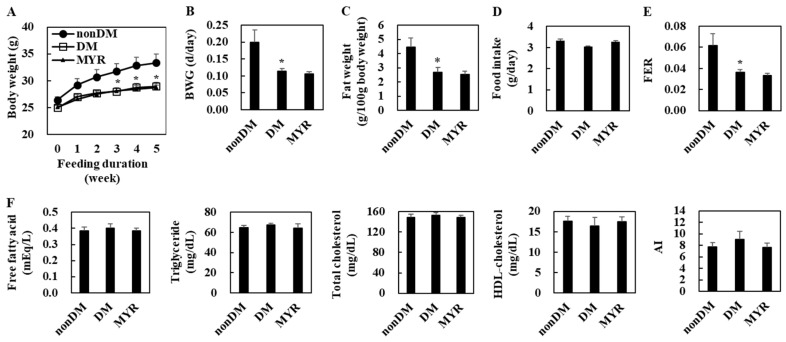
Effects of myricitrin (MYR) on change in body weight (**A**), body weight gain (**B**), fat weight (**C**), food intake (**D**), food efficiency ratio (FER) (**E**), and plasma lipids levels (**F**) in high-fat diet (HFD)/streptozotocin (STZ)-induced diabetic mice. Values are means ± SE (*n* = 10). Student’s *t*-test was used to assess the differences among groups.: * *p* < 0.05; non-DM group versus DM group. non-DM: non-diabetic group, DM: diabetic control group, MYR: MYR-supplemented diabetic group.

The body weight of the diabetic control (DM) group was significantly lower than that of the non-diabetic (non-DM) group at 3, 4, and 5 weeks of experimental diet feeding (Figure 1A). The DM group also had significantly lower body weight gain, fat weight, and food efficiency ratio (FER) than the non-DM group, although food intake did not differ between the two groups (Figure 1B–E). However, there were no significant differences in plasma lipids levels between the DM and non-DM groups (Figure 1F). In diabetic mice, MYR did not affect body weight, fat weight, food intake, and plasma lipids levels (Figure 1A–F).

### 2.2. Effects of MYR on Fasting Blood Glucose, Glucose Intolerance, Plasma Insulin, and Pancreas Immunohistochemistry

**Figure 2:**
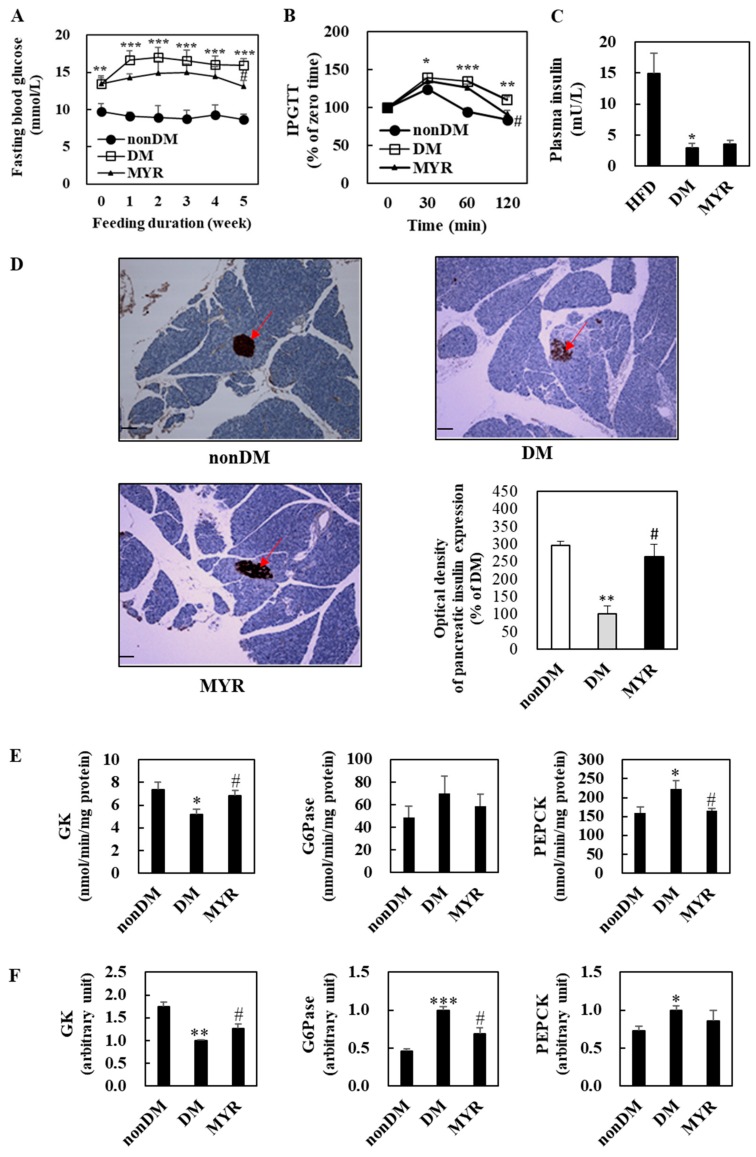
Effects of MYR on fasting blood glucose level (**A**), IPGTT (**B**), plasma insulin level (**C**), pancreas immunohistochemistry (**D**), and the hepatic glucose-regulating enzymes activities (**E**) and mRNA expression (**F**) in HFD/STZ-induced diabetic mice. (**A**–**F**): Values are means ± SE (*n* = 10). Student’s *t*-test was used to assess the differences among groups.: * *p* < 0.05, ** *p* < 0.01, *** *p* < 0.001; non-DM group versus DM group, # *p* < 0.05; DM group versus MYR group. (**D**): Representative images of immunohistochemical staining for insulin in pancreatic sections (arrows). Scale bars represent 19 µm. Magnification is 100×. IPGTT: intraperitoneal glucose tolerance test, GK: glucokinase, G6Pase: glucose-6-phosphatase, PEPCK: phosphoenolpyruvate carboxykinase.

The DM group had significantly higher fasting blood glucose levels than the non-DM group during experimental diet feeding (Figure 2A). After 5 weeks of MYR treatment, fasting blood glucose levels were significantly lower in the MYR group than in the DM group (Figure 2A). To monitor the effect of MYR on glucose homeostasis after glucose loading, we performed an intraperitoneal glucose tolerance test (IPGTT) which showed delayed glucose clearance accompanied by higher blood glucose levels in the DM group when compared to the non-DM group at 30, 60, and 120 min after glucose injection. In contrast, MYR-administered mice showed a significant decrease in blood glucose at 120 min after glucose injection (Figure 2B). Plasma insulin levels were significantly lower in the DM group than in the non-DM group, and administration of MYR slightly increased plasma insulin by approximately 20% compared to the levels in the DM group (Figure 2C). We detected insulin expression in pancreatic islets by immunohistochemical staining which revealed that the expression of insulin in the pancreatic islets was markedly lower in the DM group than in the non-DM group. The intensity of insulin staining in the islets of MYR group mice was stronger than that in islets of the DM group mice (Figure 2D). 

### 2.3. Effects of MYR on the mRNA Expression and Activities of Hepatic Glucose-Regulating Enzymes

We next investigated the mRNA expression and activities of enzymes involved in hepatic glycolysis and gluconeogenesis. Hepatic glucokinase (GK) activity and mRNA expression were significantly lower in the DM group than in the non-DM group (Figure 2E,F). In contrast, the mRNA expression and activities of glucose-6-phosphatase (G6Pase) and phosphoenolpyruvate carboxykinase (PEPCK) were markedly higher in the livers of DM group mice than in the livers of non-DM group mice (Figure 2E,F). The MYR-administered mice had significant increases in hepatic GK activity and mRNA expression, whereas the mRNA expression or activities of the gluconeogenic enzymes G6Pase and PEPCK were lower in the livers of mice in the MYR group than in the livers of mice in the DM group (Figure 2E,F).

### 2.4. Effects of MYR on Hepatic Lipids Contents, Hepatic Morphology, and the mRNA Expression and Activities of Hepatic Lipid-Regulating Enzymes 

**Figure 3:**
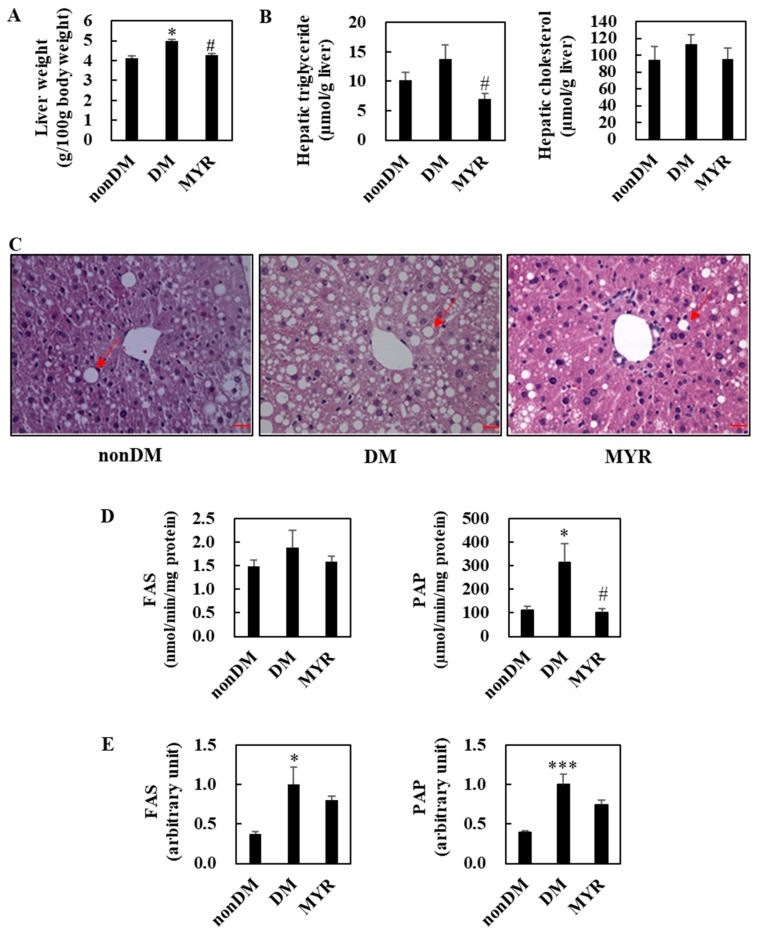
Effects of MYR on liver weight (**A**), hepatic lipids contents (**B**), liver morphology (**C**), and hepatic lipogenic enzymes activities (**D**) and mRNA expression (**E**) in HFD/STZ-induced diabetic mice. (**A**,**B**,**D**,**E**) Values are means ± SE (*n* = 10). Student’s *t*-test was used to assess the differences among groups.: * *p* < 0.05, *** *p* < 0.001; non-DM group versus DM group, # *p* < 0.05; DM group versus MYR group. (**C**) Representative images of hematoxylin and eosin stained liver sections. Arrows indicates lipid droplets. Scale bars represent 19 µm. Magnification is 400×. FAS: fatty acid synthase, PAP: phosphatidate phosphohydrolase.

The liver weights of mice in the DM group were significantly higher than those in the non-DM group, and MYR administration significantly decreased liver weight compared to that of the DM group (Figure 3A). Hepatic triglyceride content was approximately 36% higher in the DM group than in the non-DM group, and MYR administration significantly decreased hepatic triglyceride content compared to the content in the DM group (Figure 3B). MYR also reduced the diabetes-induced increase in hepatic cholesterol content, although the difference was not statistically significant (Figure 3B). Consistent with the observed changes in hepatic triglyceride content, hematoxylin and eosin staining of liver sections showed greater hepatic lipid droplet accumulation in the DM group compared to the non-DM group, whereas MYR administration markedly decreased the number and size of hepatic lipid droplets (Figure 3C). Next, we examined whether MYR could protect the liver against hepatic steatosis. We observed a 26% increase in hepatic lipogenic fatty acid synthase (FAS) activity in the DM group compared to the non-DM group, and MYR administration decreased hepatic FAS activity to levels similar to those in non-DM mice (Figure 3D). The activity of another lipogenic enzyme, phosphatidate phosphohydrolase (PAP), was also significantly higher in the DM group than in the non-DM group and was markedly decreased by MYR administration (Figure 3D). The mRNA expression levels of FAS and PAP were significantly higher in the DM group than in the non-DM group, and MYR administration decreased mRNA expression levels by 20% and 26%, respectively, compared to the levels in the DM group (Figure 3E). 

### 2.5. Effects of MYR on Plasma and Hepatic Inflammation

**Figure 4:**
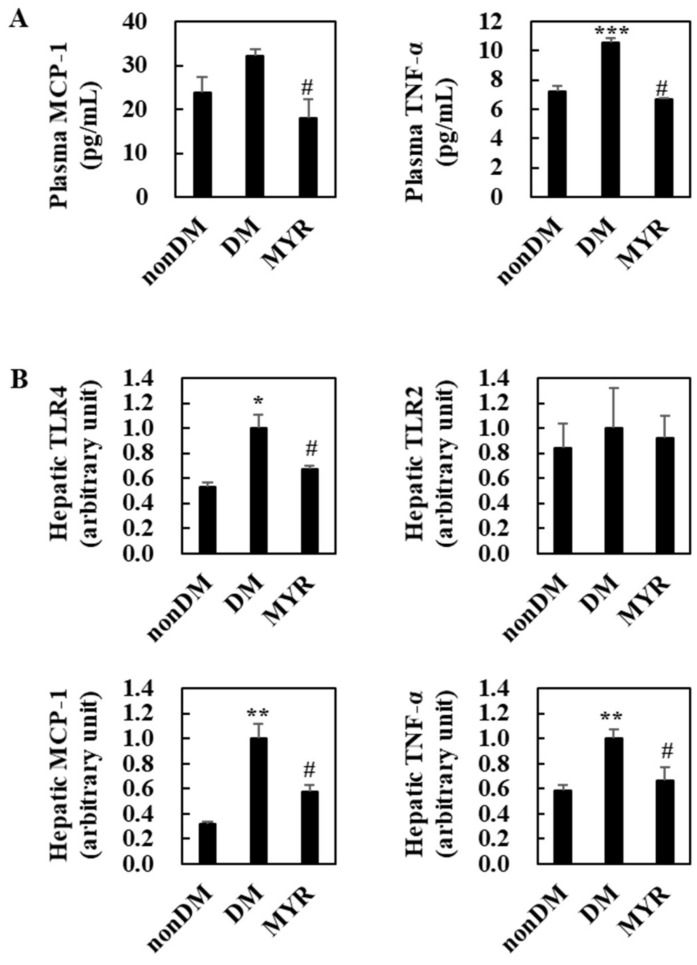
Effects of MYR on plasma pro-inflammatory MCP-1 and TNF-α levels (**A**) and hepatic pro-inflammatory genes mRNA expression (**B**) in HFD/STZ-induced diabetic mice. Values are means ± SE (*n* = 10). Student’s *t*-test was used to assess the differences among groups. * *p* < 0.05, ** *p* < 0.001, *** *p* < 0.001; non-DM group versus DM group, # *p* < 0.05; DM group versus MYR group. MCP-1: monocyte chemoattractant protein-1, TNF-α: tumor necrosis factor-α, TLR: toll-like receptor.

The DM group had significantly higher plasma and tumor necrosis factor (TNF)-α levels than the non-DM group and had somewhat higher plasma monocyte chemoattractant protein (MCP)-1 levels (Figure 4A). Administration of MYR significantly decreased plasma MCP-1 and TNF-α levels compared to the levels in the DM group (Figure 4A). The mRNA expression levels of all tested pro-inflammatory genes (toll-like receptor (TLR) 4, MCP-1, and TNF-α), except TLR2, were also significantly higher in the livers of the DM group than in the non-DM group, and MYR significantly decreased the mRNA expression of these genes in the liver compared to the levels in the DM group (Figure 4B).

## 3. Discussion

The present study demonstrated that MYR was effective for improving hyperglycemia, glucose intolerance, hepatic steatosis, and inflammation in HFD/STZ-induced diabetic mice. The anti-diabetic effects of MYR were associated with increased insulin expression in pancreatic β-cells as well as the activation of glycolysis and the inhibition of glucose production in the liver. MYR also inhibited hepatic *de novo* lipogenesis and improved inflammation.

Hyperglycemia, insulin resistance, and β-cell failure are key characteristics of type 2 diabetes. In accordance with previous findings, compared to non-DM mice, our HFD/STZ-treated mice showed higher levels of fasting blood glucose and insulin resistance as demonstrated by the impaired glucose tolerance in an IPGTT test [18,19,20]. In addition, plasma insulin levels and the numbers of pancreatic insulin-secreting β-cells were markedly decreased in the HFD/STZ-treated DM mice compared to these values in non-DM mice. These results are consistent with previous findings suggesting that administration of STZ in HFD-fed mice induces β-cell dysfunction and decreases insulin levels [18,19]. Interestingly, MYR significantly decreased fasting blood glucose levels and ameliorated glucose intolerance and pancreatic β-cell failure in HFD/STZ-induced diabetic mice, indicating that MYR may be useful agent for improving type 2 diabetes.

In diabetes, hyperglycemia is largely caused by increased hepatic glucose production and reduced glucose utilization. The rate-limiting step of glucose utilization in the liver is catalyzed by GK, which phosphorylates glucose to generate glucose 6-phosphate. After a meal, excess blood glucose is rapidly taken up by the liver. After phosphorylation by GK, glucose can no longer be exported to the circulation; consequently, it remains in the liver and facilitates hepatic glucose utilization. Therefore, GK is considered to be a key regulator of blood glucose levels and glucose tolerance [21]. It is generally accepted that GK activators are potential anti-hyperglycemic agents for the treatment of type 2 diabetes [21]. Increased hepatic gluconeogenesis also plays an important role in the pathophysiology of hyperglycemia [22]. The regulation of gluconeogenesis largely depends on the expression of gluconeogenic genes such as PEPCK and G6Pase [23,24]. In type 2 diabetic mice, such as HFD/STZ-treated mice, the mRNA levels of hepatic PEPCK and G6Pase are elevated [25], and slight changes in the expression of these gluconeogenic genes can alter blood glucose levels [26]. In the present study, MYR significantly increased the mRNA expression and activity levels of hepatic GK when compared to the levels in DM mice. Moreover, MYR normalized the hepatic mRNA and activity levels of G6Pase and PEPCK. Therefore, regulation of hepatic GK, G6Pase, and PEPCK by MYR may contribute to its blood glucose-lowering effects in HFD/STZ-treated diabetic mice.

In type 2 diabetes, insulin resistance is commonly associated with abnormalities in lipid metabolism, including enhanced *de novo* lipogenesis in the liver [27]. Although it is not known why hepatic *de novo* lipogenesis, an insulin-sensitive pathway, is activated in insulin resistance, the prevalence of NAFLD is up to three times higher in patients with type 2 diabetes compared to the general population (~90% versus 30%) [28]. Recently, Petersen et al. [1] suggested that hepatic insulin resistance caused by excessive lipid deposition in the liver precedes impairment of the insulin-mediated suppression of gluconeogenesis. Despite the controversy surrounding the cause and effect relationship between insulin resistance and hepatic steatosis, type 2 diabetes is strongly associated with NAFLD. Fujii et al. [29] demonstrated that HFD/STZ-treated mice develop NAFLD. Consistent with a previous report, in the present study, HFD/STZ-treated mice showed increased liver weight and lipid droplet accumulation compared to non-DM mice. In addition, the mRNA expression and activity levels of enzymes involved in *de novo* lipogenesis, such as FAS and PAP, were higher in HFD/STZ-treated mice than in non-DM mice. However, MYR significantly decreased liver weight as well as triglyceride contents and lipid droplet accumulation in the liver. The beneficial effect of MYR on hepatic steatosis may be related to the suppression of *de novo* lipogenesis as evidenced by the decreased hepatic lipogenic enzyme activity and gene expression levels.

Excessive lipid accumulation in the liver promotes inflammation through the release of various pro-inflammatory chemokines and cytokines, including MCP-1 and TNF-α, and these pro-inflammatory mediators can interfere with insulin signaling [30,31]. Along with these inflammatory chemokines and cytokines, perturbations in key innate immune receptors, called TLRs, have been linked with metabolic diseases such as NAFLD and type 2 diabetes. In particular, TLR4 is an important mediator of insulin resistance and hepatic inflammatory pathways [32,33]. Activation of TLR4 induces liver injury and fibrosis by triggering the production of various pro-inflammatory cytokines [33]; mice lacking hepatocyte TLR4 exhibit improved glucose intolerance, insulin sensitivity, and hepatic steatosis [34]. MCP-1, a chemoattractant protein that recruits immune cells to sites of inflammation, and TNF-α, a pro-inflammatory cytokine that inhibits insulin secretion and induces apoptosis of β-cells [35], also play pivotal roles in the development of insulin resistance and hepatic steatosis [36]. In a recent study, inhibition of C–C chemokine receptor 2, the MCP-1 receptor, ameliorated insulin resistance and hepatic steatosis by regulating hepatic lipid homeostasis in type 2 diabetic model mice [37], and anti-TNF-α treatment improved hepatic steatosis and insulin resistance [38,39]. In the present study, we demonstrated that hepatic inflammation was increased in HFD/STZ-induced type 2 diabetic mice as evidenced by the upregulation of TLR4, MCP-1, and TNF-α mRNA expression. The levels of circulating MCP-1 and TNF-α were also significantly increased in HFD/STZ-treated mice. However, MYR treatment significantly reduced plasma MCP-1 and TNF-α levels as well as the mRNA expression of hepatic TLR4, MCP-1, and TNF-α, suggesting that MYR suppresses inflammation in HFD/STZ-induced diabetic mice. 

In conclusion, the present study demonstrated that MYR exerts beneficial effects on hyperglycemia, insulin resistance, hepatic steatosis, and inflammation in HFD/STZ-induced type 2 diabetic mice by regulating glucose and lipid metabolism in the liver and protecting pancreatic β-cells. Accordingly, MYR ameliorated hyperglycemia by increasing glucose utilization and decreasing gluconeogenesis in the liver and increased pancreatic β-cell mass. In addition, MYR improved NAFLD by inhibiting *de novo* lipogenesis and inflammation in the liver, which may also be associated with the improved insulin resistance observed in MYR-administered mice. Therefore, we accepted the hypothesis that MYR protects against type 2 diabetes. Collectively, these findings suggest that MYR may be a natural bioactive compound that can improve hyperglycemia, insulin resistance, hepatic steatosis, and inflammation in type 2 diabetes. 

## 4. Materials and Methods

### 4.1. Animals and Diets

Thirty male C57BL/6J mice were purchased from Jackson Laboratory (Bar Harbor, ME, USA) at 4 weeks of age. The animals were maintained under a 12 h light–dark cycle at a constant temperature (24 ± 2 °C). They were fed a pelletized commercial chow diet for 1 week, and then the mice were randomly divided into three groups: the non-diabetic (non-DM, *n* = 10), diabetic control (DM, *n* = 10), and MYR-supplemented diabetic (MYR, *n* = 10) groups. All mice were fed a HFD (20% fat based on AIN-76 diet plus 1% cholesterol, w/w) for 4 weeks, and then the mice in the diabetic groups (DM and MYR groups) were administered STZ (100 mg kg^−1^ body weight in 0.1 M citrate buffer at pH 4.5, twice at weekly intervals, Sigma–Aldrich, St. Louis, MO, USA) through intraperitoneal injection, and continued on the HFD until diabetes was induced (at 11 weeks of age). Mice with fasting blood glucose concentrations greater than 200 mg/dL were considered diabetic and were selected for subsequent experiments. Diabetic mice were fed an HFD with or without MYR (0.005% *w*/*w*, Sigma–Aldrich, St. Louis, MO, USA) for an additional 5 weeks (until 16 weeks of age). The ingredient composition of the diets is shown in Table 1. Non-diabetic mice were fed an HFD and injected with citrate buffer. All mice had *ad libitum* access to food and water. During the experiment, food intake was monitored three times per week, and body weight was measured weekly.

At the end of the experimental period, the mice were anesthetized after a 12 h fast, and blood samples were taken from the inferior vena cava. Blood was collected in a heparin-coated tube and centrifuged at 1000× *g* for 15 min at 4 °C to separate plasma. After blood collection, the liver, pancreas, and epididymal white adipose tissue were removed, washed with physiologic saline solution, and weighed. A small piece of the liver and pancreas were fixed in 10% (*v*/*v*) formaldehyde for histological analysis, and the rest of the collected tissues were stored at −80 °C until analysis. These experiments were carried out in accordance with the recommendations of the Guide for the Care and Use of Laboratory Animals of the National Institute of Health and in accordance with the Animal Care Committee of the Pukyong National University. All procedures were approved by the Animal Ethics Committee at Pukyong National University (Approval No. 2017-45, Approval date: 10 November 2017).

### 4.2. Blood Analysis

Blood glucose concentrations were measured weekly in whole blood obtained from the tail vein after 4 h of fasting using a glucose analyzer (Accu-Chek Performa, Roche Diagnostics Unc., Indianapolis, IN, USA). An IPGTT was performed at 4 weeks (15 weeks of age) after starting the experimental diets. The mice were fasted for 4 h, and blood was obtained from the tail vein before and at 30, 60, 90, and 120 min after intraperitoneal injection of glucose (1 g kg^−1^ body weight). Blood glucose levels were measured with a glucose analyzer (Accu-Chek Performa, Roche Diagnostics Unc., Indianapolis, IN, USA). Plasma levels of insulin, MCP-1, and TNF-α were measured using a MILLIPLEX kit (Merck Millipore, Billerica, MA, USA). Plasma free fatty acid levels were determined using a commercial kit (Wako Chemicals, Osaka, Japan). Triglyceride, total cholesterol, and HDL-cholesterol levels were measured spectrophotometrically using commercial kits (Asan Pharm Co., Seoul, Korea). The atherogenic index was calculated as follows: atherogenic index = ((total cholesterol) − (HDL − cholesterol))/(HDL − cholesterol).

### 4.3. Hepatic Lipid Contents

Hepatic lipids were extracted using the procedure developed by Folch et al. [40]. Briefly, a liver sample (0.1 g) was homogenized with 1 mL of chloroform and methanol (2:1, *v*/*v*). Next, the sample was filtered through Whatman filter paper and dried under nitrogen gas at 60 °C. The dried liver sample was dissolved in 1 mL of ethanol for triglyceride and cholesterol assays. Triton X-100 and a sodium cholate solution in distilled water were mixed with 200 μL of the dissolved lipid solution for emulsification. The hepatic triglyceride and cholesterol contents were then analyzed using the same enzymatic kits used for the plasma analyses.

### 4.4. Enzyme Analyses

The enzyme fraction from the liver was prepared according to the method of Hulcher and Oleson [41] with a slight modification. The GK activity in the cytosol was determined using the spectrophotometric assay described by Davidson and Arion [42] with a slight modification, in which the formation of glucose-6-phosphate was coupled to its oxidation by glucose-6-phosphate dehydrogenase and NAD^+^ at 37 °C. The G6Pase activity was determined in the microsomes using a spectrophotometric assay according to the method of Alegre et al. [43] with a slight modification. The reaction mixture contained the following components: 100 mmol L^−1^ sodium HEPES (pH 6.5), 26.5 mmol L^−1^ glucose-6-phospate, 1.8 mmol L^−1^ EDTA, both adjusted to pH 6.5, 2 mmol L^−1^ NADP^+^, 0.6 KIU/L mutarotase, and 6 KIU/L glucose dehydrogenase. The PEPCK activity in the cytosol was monitored using the spectrophotometric assay developed by Bentle and Lardy [44] with a slight modification. Each 1 mL reaction mixture contained the following components: 50 mM sodium HEPES (pH 6.5), 1 mM IDP, 1 mM MnCl_2_, 1 mM dithiothreitol, 0.25 mM NADH, 2 mM phosphoenolpyruvate, 50 mM NaHCO_3_, 7.2 units of malic dehydrogenase, and a cytosol sample. Enzyme activity was measured at 25 °C for 2 min and was based on the decrease in the absorbance at 340 nm. The FAS activity was determined, as described by Carl et al. [45], by monitoring the malonyl-CoA-dependent oxidation of NADPH at 340 nm and was measured as oxidized NADPH in nmol/min/mg protein. The PAP activity was determined by the method of Walton and Possmayer [46]. The protein concentration was measured by the method of Bradford [47] using BSA as the standard.

### 4.5. RNA Isolation and Gene Expression Analysis

Total RNA was isolated from the liver using TRIzol reagent (Invitrogen, Grand Island, NY, USA). The isolated RNA was treated with DNase to remove contaminating DNA. The purity and integrity of the RNA were evaluated with an Agilent 2100 Bioanalyzer (Agilent Technologies, Palo Alto, CA, USA). Then, cDNA was synthesized using the PrimeScript™ RT Reagent Kit (Takara Korea Biomedical, Inc., Seoul, Korea). The mRNA expression of genes was measured by quantitative real-time polymerase chain reaction using SYBR^®^ Premix Ex TaqTM II (Takara Korea Biomedical, Inc., Seoul, Korea) and the CFX96TM real-time system (Bio-Rad, Hercules, CA, USA). The relative changes in gene expression were analyzed using the 2^−∆∆*C*t^ method, and GAPDH was used for normalization.

### 4.6. Histological and Immunohistochemical Analyses

The livers and pancreases were fixed in 10% (*v*/*v*) formaldehyde and routinely processed by washing, dehydration, clearing, paraffin embedding, casting, and sectioning into 5 µm slices. Liver tissue sections were stained with hematoxylin and eosin, and stained areas were viewed using an optical microscope (Eclipse E200; Nikon, Tokyo, Japan) at 400× magnification. Pancreatic tissue sections were stained with an anti-insulin antibody and subjected to immunohistochemistry analysis, and the stained area was visualized using an optical microscope (Eclipse E200; Nikon, Tokyo, Japan) at 100× magnification. Densitometric analysis of insulin-positive cells was performed according to previous studies with some modifications [48,49]. The optical density of insulin-positive cells in stained sections was measured using Science Lab 2001 Image Gauge 4.0 (Fuji Film, Tokyo, Japan). To control for variations caused by Mayer’s hematoxylin stain, the measured density of the other area in same section was subtracted from the measured density of insulin-stained area for each section.

### 4.7. Statistical Analyses

All data are presented as the means ± SE. Statistical analyses were performed using the Statistical Package for the Social Sciences software (SPSS) program, version 22 (SPSS Inc., Chicago, IL, USA). Student’s *t*-test was used to assess the differences among groups. *p*-Values less than 0.05 were considered statistically significant. Sample size was calculated by a G power program (version 3.1). Power analysis indicated that at least 10 animals per group were needed to detect differences in glucose and lipid metabolism in the setting of a power of 0.85 and an α probability of 0.05.

## Figures and Tables

**Table 1:** Ingredient composition of the diets fed to mice (g kg^−1^).

Ingredient (g)	HFD	MYR
Casein	200	200
DL-Methionine	3	3
Corn Starch	111	111
Sucrose	369.96	369.96
Cellulose	50	50
Corn Oil	30	30
Lard	170	170
Mineral Mixture ^1^	42	42
Vitamin Mixture ^2^	12	12
Choline Bitartrate	2	2
Cholesterol	10	10
Tert-Butylhydroquinone	0.04	0.04
Myricitrin	0	0.05

HFD, high-fat diet control; MYR, myricitrin. ^1^ AIN-76 mineral mixture (g kg^−1^): calcium phosphate 500, sodium chloride 74, potassium citrate, monohydrate 220, potassium sulfate 52, magnesium oxide 24, manganous carbonate 3.5, ferric citrate 6, zinc carbonate 1.6, cupric carbonate 0.3, potassium iodate 0.01, sodium selenite, pentahydrate 0.01, chromium potassium sulfate, dodecahydrate 0.55, sucrose, fine ground 118.03. ^2^ AIN-76 vitamin mixture (g kg^−1^): thiamin (81%) 0.6, riboflavin 0.6, pyridoxine HCl 0.7, niacin 3, calcium pantothenate 1.6, folic acid 0.2, biotin 0.02, Vitamin B_12_ (0.1% in mannitol) 1, vitamin A palmitate (500,000  IU g^−1^) 0.8, vitamin E, DL-alpha tocopheryl acetate (500  IU g^−1^) 10, vitamin D_3_, cholecalciferol (400,000  IU g^−1^) 0.25, vitamin K, MSB complex 0.15, sucrose, fine ground 981.08.

## Abbreviations

FAS	Fatty acid synthase
GK	Glucokinase
G6Pase	Glucose-6-phosphatase
HFD	High-fat diet
IPGTT	Intraperitoneal glucose tolerance test
MCP-1	Monocyte chemoattractant protein-1
MYR	Myricitrin
NAFLD	Non-alcoholic fatty liver disease
PAP	Phosphatidate phosphohydrolase
PEPCK	Phosphoenolpyruvate carboxykinase
STZ	Streptozotocin
TLR	Toll-like receptor
TNF-α	Tumor necrosis factor-α
